# The Expression of Toll-Like Receptor 4 mRNA in PBMCs Is Upregulated in Smokers and Decreases Upon Smoking Cessation

**DOI:** 10.3389/fimmu.2021.667460

**Published:** 2021-04-28

**Authors:** Hsin-Yu Yeh, Shou-Hung Hung, Su-Chiu Chen, Fei-Ran Guo, Hsien-Liang Huang, Jen-Kuei Peng, Chung-Sheng Lee, Jaw-Shiun Tsai

**Affiliations:** ^1^ Department of Family Medicine, National Taiwan University Hospital, Taipei, Taiwan; ^2^ Department of Family Medicine, College of Medicine, National Taiwan University, Taipei, Taiwan; ^3^ Department of Community and Family Medicine, National Taiwan University Hospital Yun-Lin Branch, Yun-Lin, Taiwan; ^4^ Department of Health Care Management, National Taipei University of Nursing and Health Sciences, Taipei, Taiwan; ^5^ Department of Nutrition and Health Sciences, Kainan University, Taoyuan County, Taiwan; ^6^ Center for Complementary and Integrated Medicine, National Taiwan University Hospital, Taipei, Taiwan

**Keywords:** peripheral blood mononuclear cells, smoking cessation, smokers, toll-like receptor 4, inflammation

## Abstract

**Background:**

Studies have shown *in vitro* that cigarette smoke condensate stimulates monocytes to express toll-like receptor 4 (TLR4), tumor necrosis factor-α (TNF-α), and intercellular adhesion molecule 1 (ICAM-1), and enhances their adhesion to the endothelium. However, the same effects of cigarette smoking have not been explored *in vivo*. This study is to investigate the effect of cigarette smoking and smoking cessation on their mRNA expression in human peripheral blood mononuclear cells (PBMCs).

**Methods:**

A group of 97 smokers and 62 nonsmokers were enrolled. The RNA from PBMCs was assessed with real-time polymerase chain reaction (PCR) to determine the levels of ICAM-1, TNF-α, and TLR4. The same markers in PBMCs of 87 quitters were examined before and at one week, one month, and two months after smoking cessation.

**Results:**

Of the 97 smokers, 85 (87.6%) were males, and 30 (48.4%) of the nonsmokers were males (*p* < 0.0001). The mean (SD) age of the smokers was 43.24 (10.89) years, which was younger than 43.45 (11.41) years of nonsmokers (*p* < 0.0001). The incidence of cardiovascular diseases was 13.4% in smokers, which was higher than 1.6% in nonsmokers (*p* < 0.05). Both ICAM-1 and TNF-α mRNA levels in PBMCs were higher among the smokers (*p* < 0.0001). In addition, TLR4 mRNA levels in PBMCs were statistically elevated in the smokers (*p* < 0.0001) comparing with those in the nonsmokers. The mRNA levels of TLR4 and TNF-α in PBMCs decreased in those who had quit smoking for 2 months (*p* < 0.0001).

**Conclusions:**

ICAM-1, TNF-α, and TLR4 mRNA expression levels in PBMCs increased in smokers and decreased after being on a smoking cessation program for 2 months. This finding suggested that TLR4 expression may mediate the atherogenic inflammatory process induced by smoking.

## Introduction

Cigarette smoking is a major lifestyle risk factor for atherosclerosis and cardiovascular diseases, regarded as one of the biggest threats to health worldwide. The meta-analysis (1) included 141 cohort studies reported that even smoking only a single cigarette daily increased the risk of coronary artery disease and stroke. People who smoked just one cigarette daily carried 40–50% of the increased cardiovascular disease risk of those who smoked 20 cigarettes daily (1). The study in Japan (2) showed that smoking advanced carotid atherosclerosis as estimated on carotid intima-media thickness and increased the risk of atherothrombotic events such as acute myocardial infarction and stroke in patients with metabolic syndrome. Several studies also delineated that smoking had detrimental effects on arterial stiffness and pulse-pressure amplification (3, 4).

In contrast, smoking cessation gives rise to enormous health benefits for quitters of all ages in extending lifespan and significantly reducing risks of myocardial infarction (5). However, it remains unclear how smoking causes atherosclerosis, and the mechanism which improves the cardiovascular conditions after smoking cessation is also poorly understood.

Toll-like receptors (TLRs) are the most well-known family of pattern recognition receptors that initiate adaptive immune responses (6). TLRs activation is seen as a crucial step for mounting a series of forceful immune reactions against invading pathogens. However, activation of TLRs can also cause overproduction of inflammatory cytokines and chemokines. For example, long-term exposure to nicotine has been shown to upregulate the mRNA and protein levels of TLR4 and TLR6 in the epithelium and smooth muscle layer, which induced airway inflammation and hypersensitivity (7). Cigarette smoke significantly enhanced the expression of TLR2/TLR4 mRNA in histamine and lipopolysaccharide (LPS) treated cells, which suggested that upregulation of TLR2/TLR4 signaling promoted progression of atherosclerosis (8). When cultured peripheral blood mononuclear cells (PBMCs) were treated with cigarette smoke extract, the expression of TLR4 mRNA increased in a dose-dependent manner due to enhanced production of inflammatory cytokines such as interleukin 8 (IL-8) and tumor necrosis factor-α (TNF-α) (9). These findings suggest that the expression of TLR4 mRNA by monocytes/macrophages may be stimulated by cigarette smoke and may be involved in the pathogenesis of atherosclerosis.

Inflammation has been known to constitute an essential element in atherogenesis and the adhesion of mononuclear cells to the damaged endothelium plays a critical step in triggering atherosclerosis (10). Compared to non-tobacco consumers, smokers exhibited higher plasma inflammatory protein expression of intercellular adhesion molecule 1 (ICAM-1)and vascular endothelial growth factor (VEGF) (11). An *in vitro* study in 1994 delineated the possible mechanism that cigarette smoking increased the incidence of atherosclerotic diseases by enhancing the expression of the adhesive ligand CD11b on the surface of human monocytes and its receptors such as ICAM-1 in human umbilical vein endothelial cells (HUVECs), resulting in the adherence of cigarette smoke treated monocytes to HUVECs (12). A clear dose-dependent relationship between smoke intake and the serum level of soluble ICAM-1 was also identified in a clinical study (13).

Florin et al. assessed the difference of the serum inflammatory markers between smokers and nonsmokers and demonstrated high serum levels of TNF-α and C-reactive protein (CRP) in smokers (14). Poussin et al. further elucidated that aqueous cigarette smoke extract induced the adhesion of monocytic cells to HUVECs and this effect was driven mainly by TNF-α produced by monocytic cells (15).

TLR4 plays a central role in lipopolysaccharide (LPS) signaling and is necessary for macrophage differentiation in to foam cells. Experimental evidence demonstrated that both TLR4 mutation and a TLR4 blocking antibody reduced foam cell formation in the presence of oxidized lipoprotein (16). The study further showed that TLR4, treated with LPS, promoted foam cell formation (16). TLR4 also regulates the expression of TNF-α and ICAM-1. Song et al. found that ICAM-1 levels in human aortic valve interstitial cells increased after LPS stimulation, and this increase was significantly attenuated by prior treatment with TLR4-neutralizing antibody (17). Another study observed that myocardial ICAM-1 levels were significantly reduced in TNF-α knockout mice (18). Both TLR4 mutation and TNF-α knockout markedly attenuated LPS-induced myocardial ICAM-1 expression, suggesting that TNF-α signaling is involved in the regulation of myocardial ICAM-1 expression by TLR4 (18).

To date, the effect of cigarette smoking and smoking cessation on the expression of TLR4, ICAM-1, and TNF-α has not been explored. The aim of our study is to investigate the effect of cigarette smoking and smoking cessation on the mRNA expression of TLR4, ICAM-1, and TNF-α in PBMCs obtained from human subjects.

## Materials and Methods

### Subjects

Smokers aged 18 years or older were recruited from the smoking cessation clinic of the Department of Family Medicine at the National Taiwan University Hospital, Taipei, Taiwan. The inclusion criteria were as follows: (1) subjects who smoked 10 or more cigarettes per day; or (2) subjects who smoked less than 10 cigarettes per day but had a Fagerström test for nicotine dependence (FTND) score of 5 points or higher. Overall, 97 subjects were recruited from September 2007 to September 2010. The study also recruited 62 nonsmoking volunteers who received annual physical examination at the same outpatient clinic and were comparable with smokers in terms of most demographic variables. All participants did not have malignancy, acute or chronic infection. This study was approved by the institutional ethics committee (registration number: 9361701201), and written informed consent was obtained from each participant.

### Data Collection and Follow-ups

Demographic characteristics and smoking history, including the duration of smoking, the number of cigarettes smoked per day, smoking index (number of cigarettes smoked per day × number of years for which the participant had been smoking) were recorded. Chronic diseases, such as hypertension, diabetes, hyperlipidemia, cardiovascular diseases, obesity, and liver or renal diseases among participants were identified and recorded.

### Blood Sampling

Blood samples (10 mL) were drawn from all subjects at the first visit. For smokers, blood samples were also collected at 1 week, 1 month, and 2 months during the smoking cessation period. Of the 97 smokers, 87 quitters’ samples were collected.

### Isolation of Human Peripheral Mononuclear Cells and Quantitative Real-Time Polymerase Chain Reaction Analysis

Human PBMCs were isolated by centrifugation on a Ficoll sodium metrizoate density gradient (Amersham Biosciences, Uppsala, Sweden) according to the manufacturer’s instruction. Total RNA was extracted by REzol (Protech Technology, Sparks, NV) according to the manufacturer’s instruction and then used for cDNA synthesis with a high-capacity RNA-to-cDNA Kit (Applied Biosystem, Carlsbad, CA). The cDNAs were amplified in a 20 μL reaction mixture containing TaqMan gene expression master mix (Applied Biosystem, Carlsbad, CA) according to the manufacturer’s instruction. The mRNA levels of TLR4, ICAM-1, and TNF-α in PBMCs were examined. Quantitative real-time polymerase chain reaction (PCR) was performed using the ABI 7000 real-time PCR system with primers for measuring TLR4 (forward: 5’- AGTCA AGGAA CCCAT GACAA -3’, reverse: 5’- GAGAA TGACC AGGAT GGTTG -3’), ICAM-1 (forward: 5’- CAGCC AGTGG GCAAG AACCT -3’, reverse: 5’- CAGTG CGGCA CGAGA AATTG -3’), TNF-α (forward: 5’-CCCTG GTATG AGCCC ATCTA TC-3’, reverse: 5’-AAAGT AGACC TGCCC AGACT CG-3’). The cDNA concentration in each sample was normalized using transcripts of GAPDH (forward: 5’- GAAGG TGAAG GTCGG AGTC -3’, reverse: 5’- GAAGA TGGTG ATGGG ATTC -3’). The mRNA expression in PBMCs was expressed as a relative ratio by normalizing the mRNA expression of each participant to that of a nonsmoker control.

### Statistical Analyses

Descriptive statistics including frequency, percentage, median, mean, standard deviation (SD), and standard error (SE) were reported for relevant variables. Baseline data between the smokers and the nonsmokers were compared by Fisher’s exact tests (for categorized variables), independent 2-sample t-tests, or non-parametric Wilcoxon rank sum tests. The strength of linear association between 2 non-categorical variables was estimated by the Pearson correlation coefficient (r), and the null hypothesis of zero correlation (that is, the standardized slope in this setting) was tested by performing a simple linear regression analysis. To adjust for the variation in baseline demographic characteristics, multiple linear regression analysis was also utilized. Repeated measures of analysis of variance (ANOVA) were applied to compare the mRNA levels of TLR4, ICAM-1, and TNF-α in PBMCs at the first visit (baseline day 0), one week, one month, and two months after smoking cessation. The statistical software SAS version 9 (SAS Institute Inc., Cary, NC) was used for data management and analysis. The statistical significance level was set as 0.05, and all statistical tests were 2-tailed.

## Results

### Demographic Characteristics of the Study Subjects

Of the 97 smokers, 85 (87.6%) were male. The mean (SD) age of the smokers was 43.24 (10.89) years and they smoked 24.38 (12.11) cigarettes/day. Compare to the smokers, there was high percentage of female 32 (51.6%) among nonsmokers and their mean (SD) age was 43.45 (11.41) years, which is older than smokers. The smokers were highly dependent on nicotine, as indicated by a high mean FTND score of 6.33 (2.38). The mean smoking duration was 23.79 (10.87) years. Gender distribution and the presence of cardiovascular diseases were significantly different between the 62 nonsmokers and 97 smokers. Meanwhile, body mass index (BMI), and the presence of hypertension, hyperlipidemia, obesity, and diabetes mellitus were not significantly different between these two groups (**Table 1**).

**Table 1:** Baseline demographic data (n = 159).

Variable	Values [median]		n (%) or Mean (SD)	
	Nonsmokers n = 62	Smokers n = 97	Nonsmokers n = 62	Smokers n = 97
Gender				
Male			30 (48.4%)	85 (87.6%)
Female			32 (51.6%)	12 (13.4%)^**^
Age (years)	23~69 [42]	22~80 [42]	43.45 (11.41)	43.24 (10.89)^**^
Age group (years)				
22~30			9 (14.5%)	10 (10.3%)
31~45			27 (43.6%)	54 (55.7%)
46~60			23 (37.1%)	26 (26.8%)
61~80			3 (4.8%)	7 (7.2%)
Cigarettes/day	0 [0]	4~60 [20]	0 (0)	24.38^a,b^ (12.11)^**^
Smoking duration (years)	0 [0]	3~60 [22]	0 (0)	23.79^a,b^ (10.87)^**^
Smoking index	0 [0]	30~2340 [500]	0 (0)	590.34^a,b^ (410.72)^**^
= (Cigarettes/day) × years				
FTND score (0~10)	0 [0]	1~10 [6]	0 (0)	6.33^a,b^ (2.38)^**^
Body mass index (kg/m^2^)	18.90~32.00	17.63~34.56	23.83 (2.99)	24.72 (3.39)
	[23.84]	[25.06]^*^		
Obesity				
No			54 (87.1%)	73 (75.3%)
Yes			8 (12.9%)	24 (24.7%)
Cardiovascular diseases				
No			61 (98.4%)	84 (86.6%)
Yes			1 (1.6%)	13 (13.4%)^*^
Hypertension				
No			57 (91.9%)	81 (83.5%)
Yes			5 (8.1%)	16 (16.5%)
Diabetes mellitus				
No			61 (98.4%)	92 (94.8%)
Yes			1 (1.6%)	5 (5.2%)
Hyperlipidemia				
No			54 (87.1%)	87 (89.7%)
Yes			8 (12.9%)	10 (10.3%)

The other demographic nonsmoking variables (not shown) did not have statistically significant difference (p > 0.05) when the values for the nonsmokers and smokers were compared by parametric and nonparametric methods.

a Variable did not show approximately normal distribution with the observed data.

b Statistically significant unequal variances between 2 groups.

^*^p < 0.05.

^**^p < 0.0001.

FTND, Fagerström test for nicotine dependence; ICAM-1, intercellular adhesion molecule.

TNF-α, tumor necrosis factor-α; TLR4, Toll-like receptor 4.

### ICAM-1, TNF-α and TLR4 mRNA Levels in the PBMCs of the Study Subjects

We assessed the mrna levels of TNF-α, ICAM-1, and TLR4 with real-time PCR. The mean mrna levels of ICAM-1 and TNF-α in PBMCs were significantly higher among the smokers in comparison to the nonsmokers (*p* < 0.0001). The mrna levels of TLR4 in PBMCs were also statistically elevated in the smokers compared with that of the nonsmokers (*p* < 0.0001) (**Table 2**).

**Table 2:** Comparisons of mononuclear cell markers in smokers (n = 97) and nonsmoker controls (n = 62).

Variable	Group				*t*	*p* for *t* test	*p* ^c^
	Nonsmokers		Smokers				
	n	Mean (SD)	n	Mean (SD)			
ICAM-1^b^	57	0.90 (0.32)	94	1.90 (0.57)	−13.70	**<0.0001**	**<0.0001**
TNF-α^b^	57	1.14 (0.40)^a^	95	2.51 (0.79)^a^	−14.22	**<0.0001**	**<0.0001**
TLR4^b^	57	1.08 (0.46)	94	1.84 (0.76)^a^	−7.60	**<0.0001**	**<0.0001**

Statistically significant results (p < 0.05) are presented in boldface.

a Variable did not show approximately normal distribution with the observed data, but t test may be valid when n > 30 for this group.

b Statistically significant unequal variances between 2 groups; therefore, t and p values were adjusted by the Satterthwaite method.

c The Wilcoxon rank-sum test.

ICAM-1, intercellular adhesion molecule; TNF-α, tumor necrosis factor-α; TLR4, Toll-like receptor 4.

### Clinical Variables Correlated With ICAM-1, TNF-α and TLR4 mRNA Levels in the PBMCs

Simple linear regression analysis was performed to explore what clinical variables might be correlated with the mRNA levels of ICAM-1, TNF-α, and TLR4 in PBMCs.

Among the total of 159 participants, ICAM-1 mRNA levels in PBMCs positively correlated with the gender, number of cigarettes smoked per day (*p* < 0.0001), smoking index (*p* < 0.0001), smoking duration (*p* < 0.0001), level of nicotine dependence (*p* < 0.0001), and the presence of cardiovascular diseases (*p* = 0.0313) (**Table 3**). TNF-α mRNA levels tended to be more strongly correlated with the presence of cardiovascular diseases (*p* = 0.0186), hypertension (*p* = 0.0438), and obesity (*p* = 0.0034) (**Table 3**). TLR4 mRNA levels in PBMCs positively correlated with the number of cigarettes smoked per day, smoking index, smoking duration, the number of cigarettes smoked per day, and level of nicotine dependence (all *p values* < 0.0001) (**Table 3**).

**Table 3:** Simple linear regression analysis for mononuclear cell markers (n = 159).

Independent variable	Dependent variable: Mononuclear cells						
	ICAM-1 (n=151)		TNF-α (n=152)		TLR4 (n=151)		
	Stand. slope^a^ (*p*)		Stand. slope^a^ (*p*)		Stand. slope^a^ (*p*)		
Age (years)	−0.0258	(0.7532)	−0.0144	(0.8602)	−0.0780	(0.3413)	
Gender (0: Male, 1: Female)	−0.3626	(**<0.0001**)	−0.3390	(**<0.0001**)	−0.3069	(**0.0001**)	
Body mass index (kg/m^2^)	0.0803	(0.3272)	0.1380	(0.0900)	0.1405	(0.0853)	
Group							
(0: Nonsmokers, 1: Smokers)	0.7380	(**<0.0001**)	0.7964	(**<0.0001**)	0.5156	(**<0.0001**)	
Cigarettes/day	0.5724	(**<0.0001**)	0.5855	(**<0.0001**)	0.3962	(**<0.0001**)	
Smoking duration (years)	0.5965	(**<0.0001**)	0.6430	(**<0.0001**)	0.4075	(**<0.0001**)	
Smoking index	0.4948	(**<0.0001**)	0.5004	(**<0.0001**)	0.3305	(**<0.0001**)	
FTND score	0.6438	(**<0.0001**)	0.6738	(**<0.0001**)	0.4901	(**<0.0001**)	
Cardiovascular diseases							
(0: No, 1: Yes)	0.1753	(**0.0313**)	0.1907	(**0.0186**)	0.1253	(0.1252)	
Hypertension (0: No, 1: Yes)	0.0597	(0.4665)	0.1637	(**0.0438**)	0.0488	(0.5519)	
Diabetes mellitus (0: No, 1: Yes)	0.0101	(0.9016)	0.0048	(0.9533)	0.0377	(0.6460)	
Hyperlipidemia (0: No, 1: Yes)	−0.0185	(0.8220)	0.0362	(0.6579)	0.0739	(0.3670)	
Obesity (0: No, 1: Yes)	0.1527	(0.0613)	0.2365	(**0.0034**)	0.1479	(0.0699)	

Statistically significant results (p < 0.05) are presented in boldface. The transformation of natural logarithm was performed on the dependent variable.

a Estimate of standardized slope, that is, estimate of the Pearson correlation coefficient, r, in this setting.

FTND, Fagerström test for nicotine dependence; ICAM-1, intercellular adhesion molecule.

TNF-α, tumor necrosis factor-α; TLR4, Toll-like receptor 4.

In the multiple linear regression analysis, after controlling for the demographic and clinical variables, smoking remained the single independent factor affecting TLR4 and ICAM-1 mRNA levels in PBMCs (all *p* values < 0.0001) (**Table 4**). Age (*p* = 0.00217) was another independent factor affecting TNF-α mRNA levels in PBMCs (**Table 4**).

**Table 4:** Multiple regression analysis of each main outcome-dependent variable for mononuclear cell markers (n = 159).

Independent variables	Dependent variable: Mononuclear cells		
	ICAM-1 (n=151)	TNF-α (n=152)	TLR4 (n=151)
	Stand. slope^a^ (*p*)	Stand. slope^a^ (*p*)	Stand. slope^a^ (*p*)
Model 1: adjusted for age			
Group 0: Nonsmokers	0	0	0
1: Smokers	0.74 (**<0.0001**)	0.80 (**<0.0001**)	0.52 (**<0.0001**)
Model 2: adjusted for age, gender			
Group 0: Nonsmokers	0	0	0
1: Smokers	0.71 (**<0.0001**)	0.80 (**<0.0001**)	0.47 (**<0.0001**)
Model 3: adjusted for age, gender, BMI			
Group 0: Nonsmokers	0	0	0
1: Smokers	0.71 (**<0.0001**)	0.80 (**<0.0001**)	0.47 (**<0.0001**)
Model 4			
Group: Nonsmokers (0), smokers (1,2)	0.69 (**<0.0001**)	0.76 (**<0.0001**)	0.49 (**0.0001**)
(1: Smoking years ≤ 15, median)			
(2: Smoking years > 15)			
Age ≥ 42 years (median) 0: No	0	0	0
1: Yes	−0.09 (0.1701)	−0.19 (**0.0017**)	−0.06 (0.4169)
Gender 0: Male	0	0	0
1: Female	−0.06 (0.3628)	0.02 (0.7454)	−0.07 (0.4223)
Body mass index (BMI) (kg/m^2^)	−0.04 (0.5261)	−0.01 (0.9241)	0.04 (0.6250)
Cardiovascular diseases 0: No	0	0	0
1: Yes	0.03 (0.6789)	0.01 (0.9318)	<0.01 (0.9979)
Hypertension 0: No	0	0	0
1: Yes	−0.02 (0.8007)	0.11 (0.0711)	−0.03 (0.7380)
Diabetes mellitus 0: No	0	0	0
1: Yes	−0.03 (0.6068)	−0.06 (0.3108)	−0.02 (0.8173)
Hyperlipidemia 0: No	0	0	0
1: Yes	<0.01 (0.9988)	0.06 (0.2649)	0.08 (0.3115)

Statistically significant results (p < 0.05) are presented in boldface. The transformation of natural logarithm was performed on the dependent variable.

a Estimate of standardized slope.

ICAM-1, intercellular adhesion molecule; TNF-α, tumor necrosis factor- α; TLR4, Toll-like receptor 4.

### Effect of Smoking Cessation on ICAM-1, TNF-α and TLR4 mRNA Levels in PBMCs

After two months of follow-up, our results showed significant decreases in the mRNA levels of TNF-α and TLR4 in PBMCs during the smoking cessation period (*p* < 0.0001) (**Table 5**). There was also a decreasing tendency in ICAM-1 mRNA levels during the smoking cessation period, although the difference did not reach statistical significance.

**Table 5:** Comparisons of mononuclear cell markers in 87 smokers stop smoking (excluding 10 non-quitters) with repeated measures during follow-up.

Variables	BaselineDay 0,	One week,	1 month,	2 months,	*p* ^e^	*p* ^f^for trend
	n = 87	n = 56	n = 33	n = 21		
	Mean (SE)	Mean (SE)	Mean (SE)	Mean (SE)		
ICAM-1	1.91 (0.59)^b^	1.73 (0.52)^d^	1.85 (0.74)	1.67 (0.80)	0.0899	0.0934
TNF-α	2.46 (0.78)^ac^	2.30 (0.51)^d^	2.25 (0.49)	2.08 (0.58)	**0.0010**	**<0.0001**
TLR4	1.83 (0.80)^ab^	1.54 (0.49)^d^	1.56 (0.67)	1.32 (0.63)	**<0.0001**	**<0.0001**

Statistically significant results (p < 0.05) are presented in boldface.

a Variable did not show approximately normal distribution with the observed data, but the changes of repeated measures did, so repeated measures ANOVA is a valid analysis.

b Three missing values.

c Two missing values.

d One missing value.

e p values are based on the general linear model for 4 repeated measures ANOVA, degrees of freedom (df) = (3, 106).

f p values are based on the general linear model for repeated measures ANOVA by considering quit intervals (1, 2, 3, 4) trend, df = (1, 108).

SE, standard error; ICAM-1, intercellular adhesion molecule; TNF-α, tumor necrosis factor-α ;TLR4, Toll-like receptor 4.

## Discussion

Smoking has been linked to increased atherosclerosis and TLR4 has been viewed as a contributor to the pathogenesis of atherosclerosis (6). Our study for the first time demonstrates that TLR4 mRNA expression levels in PBMCs in smokers are higher than that in nonsmokers. TLR4 mRNA expression levels in PBMCs were positively correlated with the number of cigarettes smoked per day, smoking index, smoking duration, and level of nicotine dependence. Furthermore, TLR4 mRNA levels in PBMCs decreased significantly in 2 months after smoking cessation.

Our data showed that there is higher percentage of males among smokers than nonsmokers. National Health Interview Survey (NHIS) of Taiwan conducted in 2001 elucidated that Taiwan has particularly high male smoking prevalence and much lower female smoking prevalence (46.8% *vs* 4.3%), which have played an important role in the shortening in life expectancy observed among males in Taiwan (19). Age distribution was also different between smokers and nonsmokers. Therefore, we adjusted for sex and age in the multiple regression analysis and showed that smoking was still an independent factor for TLR4 mRNA expression in PBMCs.

There is mounting evidence that TLR4 triggers the initial lung inflammation in chronic obstructive pulmonary disease (COPD). An exploratory *in vitro* experiment performed by Khalil et al. showed that after cigarette smoke exposure, monocytes were the major source of IL-8 (20). Furthermore, pretreatment of monocytes with anti-human TLR4 antibody markedly blocked the IL-8 generation in PBMCs, suggesting that TLR4 may be involved in the activation of cytokine production that links smoking with inflammation in COPD. Our study shows that TLR4 mRNA expression levels were higher in PBMCs in smokers than nonsmokers and its levels in PBMCs decreased significantly in 2 months after smoking cessation. Together, these data support the possibility that TLR4 plays a role in the inflammatory process initiated by cigarette smoking.

In addition to TLR4, both ICAM-1 and TNF-α are well known inflammatory markers and have been extensively studied biomarkers known to contribute to systemic inflammation in smokers (13, 15, 21). In a cross-sectional study, a significantly reduction of circulating serum TNF-α, which is a predictive factor of cardiovascular diseases, was observed among participants over the course of a smoking cessation program (21). Another study in 2007 reported that soluble ICAM-1 concentration in the plasma decreased during a 1-year follow-up in quitters with cardiovascular risk factors (22). TNF-α and ICAM-1 mRNA levels in PBMCs were collected in a small number of the participants (6 quitters and 6 smokers) but did not show significant differences at baseline and 1 year later possibly due to the small sample size (22). In the present study, we directly assessed the changes in the mRNA expression levels of ICAM-1 and TNF-α in PBMCs rather than in serum. Our data demonstrated that ICAM-1 and TNF-α mRNA levels in PBMCs were significantly higher among the smokers. Meanwhile, TNF-α mRNA expression levels in PBMCs significantly decreased 2 months after smoking cessation in 87 quitters. ICAM-1 mRNA expression levels also showed an insignificant decreasing trend in PBMCs after 2 months of smoking cessation. These results may help explain the anti-inflammatory effects of smoking cessations.

Multiple pro-inflammatory mediators contribute to cardiac dysfunction caused by bacterial LPS. Current research in the pathogenesis of calcific aortic valve stenosis showed that cellular ICAM-1 levels increased 10-fold after 24h of LPS stimulation, and this increase was remarkably attenuated by prior treatment with TLR4-neutralizing antibody, suggesting that TLR4 plays a critical role in mediating the ICAM-1response to LPS in human aortic valve interstitial cells (17). In accordance with previous investigations, the study by Ao et al. illustrated that TLR4 mutation abolished cellular ICAM-1expression correlated with an abrogation of LPS-induced TNF-α production (18). In the present study, TLR4 expression in PBMCs may be a pivotal biomarker linking cigarette smoking to inflammatory cytokine production and inflammatory process.

An *in vitro* study conducted by Giunzioni et al. revealed that cigarette smoke condensate augmented the TNF-α, IL-8 and TLR4 mRNA expression in monocytes (23). The pre-treatment of monocytes with inhibitor of NF-κB completely counteracted the cigarette smoke condensate stimulatory effect on TNF-α and IL-8 expression, further suggesting that a role for NF-κB signaling in inflammatory cytokine production. A recent study also established that LPS significantly initiated the TLR4/Myeloid differentiation primary response 88 (MyD88) pathway, which in turn induced the activation of NF-κB and ultimately induces ICAM-1 expression in human pulmonary alveolar epithelial cells which may contribute to the inflammatory responses in various lung diseases (24). The aforementioned results suggested that inflammatory cascades may be initiated by the TLR4 pathway *via* NF-κB signaling activation.

Our present study has some limitations. First, since this was an observational study, no causal relationship could be determined. Second, the results we showed in the present study were based on mRNA expression, which may not correlate with the degrees of difference at the protein levels. Future studies are required to investigate the changes at the protein levels after smoking cessation. Third, we did not examine the expression of other Toll-liker receptors, such as TLR2, which is also implicated in the atherogenesis. Fourth, we need to extend our follow-up duration to verify the long-term effect of smoking cessation on TLR4 mRNA expression levels and the clinical manifestation of the associated cardiovascular diseases.

In the future, we may need to conduct large-scale prospective cohort studies to explore whether smoking cessation, with decreased the levels of TLR4 mRNA expression in PBMCs, will lead to a significant reduction in the risk of cardiovascular disease and the associated mortality.

In conclusion, TLR4, ICAM-1, and TNF-α mRNA expression levels in PBMCs were higher in smokers than nonsmokers, and decreased significantly after 2-months of smoking cessation. Although the exact mechanism of how smoking cessation reduces the risk of cardiovascular events has not been well explored, TLR4 levels in monocytes seem to play an important role. Our finding suggested that TLR4 may mediate the inflammatory process of atherosclerosis in smokers and smoking cessation may have an anti-inflammatory effect by reducing the level of TLR4 and the production of inflammatory cytokines. The detailed mechanism warrants further investigation.

## Data Availability Statement

The original contributions presented in the study are included in the article/supplementary material. Further inquiries can be directed to the corresponding author.

## Ethics Statement 

The studies involving human participants were reviewed and approved by Institutional ethics committee. The patients/participants provided their written informed consent to participate in this study.

## Author Contributions

H-YY, S-HH, and J-ST were responsible for the study concept and design. F-RG, H-LH, and J-KP contributed to the acquisition of clinical data. H-YY, S-HH, S-CC, C-SL, and J-ST assisted with data analysis and interpretation of findings. H-YY drafted the manuscript. H-YY, S-HH, and J-ST provided critical revision of the manuscript for important intellectual content. All authors contributed to the article and approved the submitted version.

## Funding

This work was supported by the grant (NSC 98-2314-B-002 -118 -MY2, NSC 101-2314-B-002 -042, NSC 102-2314-B-002 -031, and MOST 103-2314-B-002 -129 to J-ST) from the National Science Council, Taiwan. The financial sponsors played no role in any aspect of the study.

## Conflict of Interest

The authors declare that the research was conducted in the absence of any commercial or financial relationships that could be construed as a potential conflict of interest.
